# Plasmatic membrane toll-like receptor expressions in human astrocytomas

**DOI:** 10.1371/journal.pone.0199211

**Published:** 2018-06-18

**Authors:** Isabele Fattori Moretti, Daiane Gil Franco, Thais Fernanda de Almeida Galatro, Sueli Mieko Oba-Shinjo, Suely Kazue Nagahashi Marie

**Affiliations:** Laboratory of Molecular and Cellular Biology (LIM 15), Department of Neurology, Faculdade de Medicina FMUSP, Universidade de Sao Paulo, Sao Paulo, Brasil; Northern University, UNITED STATES

## Abstract

Toll-like receptors (TLRs) are the first to identify disturbances in the immune system, recognizing pathogens such as bacteria, fungi, and viruses. Since the inflammation process plays an important role in several diseases, TLRs have been considered potential therapeutic targets, including treatment for cancer. However, TLRs’ role in cancer remains ambiguous. This study aims to analyze the expression levels of plasmatic cell membrane TLRs (TLR1, TLR2, TLR4, TLR5, and TLR6) in human astrocytomas the most prevalent tumors of CNS different grades (II-IV). We demonstrated that TLR expressions were higher in astrocytoma samples compared to non-neoplastic brain tissue. The gene and protein expressions were observed in GBM cell lines U87MG and A172, proving their presence in the tumor cells. Associated expressions between the known heterodimers TLR1-TLR2 were found in all astrocytoma grades. In GBMs, the mesenchymal subtype showed higher levels of TLR expressions in relation to classical and proneural subtypes. A strong association of TLRs with the activation of cell cycle process and signaling through canonical, inflammasome and ripoptosome pathways was observed by *in silico* analysis, further highlighting TLRs as interesting targets for cancer treatment.

## Introduction

For the past decade, the inflammatory response in the tumor microenvironment has been in the spotlight due to the controversy regarding its ultimate effect. Despite the expectation that inflammation would wield a positive effect against tumorigenesis, relevant studies about the tumor-promoting effects of immune cells have been published. The resulting bioactive molecules from inflammation can provide the tumor microenvironment with growth factors that sustain proliferative signaling, with survival factors that impair apoptosis, along with proangiogenic and extra-cellular matrix-remodeling factors that facilitate invasion, metastasis, and the formation of new blood vessels [1].

Toll-like receptors (TLRs) act as the first line of defense, recognizing nonself-molecules and activating inflammatory processes [2]. Therefore, TLRs have been considered as potential targets for tumor therapeutics [3, 4].

There are two subgroups of TLRs, based on the subcellular location of these proteins and their respective pathogen-associated molecular pattern (PAMP) ligands. In humans, cell surface TLR1, TLR2, TLR4, TLR5, and TLR6 comprise one group, recognizing mainly microbial membrane components such as lipids, lipoproteins, and proteins. The other group consists of TLR3, TLR7, TLR8, and TLR9, which are expressed exclusively in intracellular vesicles, including endoplasmic reticulum (ER), endosomes, lysosomes, and endolysosomes, and recognizing microbial nucleic acids. TLR2 and TLR4 also identify endogenous molecules rising upon tissue injury, the damage-associated molecular patterns (DAMPs), such as heat-shock proteins, including HSP70, HSP60 Gp96, HSP22, and HSP72, and high-mobility group box-1, protein, as well as extra-cellular matrix (ECM) molecules such as biglycan, tenascin-C, versican, and fragments of ECM molecules (oligosaccharides of hyaluronic acid and heparan sulfate) [5].

Structurally, TLRs are integral membrane receptors, presenting a N-terminal ligand recognition domain, with a leucine-rich repeat motif, a single transmembrane helix, and a C-terminal cytoplasmic signaling domain—known as Toll IL-1 receptor (TIR) domain due to the homology of signaling domains of IL-1R family members [6].

TLR1, TLR2, TLR4, TLR5, and TLR6 are involved in a diversity of cellular responses, ranging from cell proliferation to cell death, and as such they are the targets of the current study in astrocytomas. Cell surface TLRs may present distinct signaling pathways. Below is a brief summary of their signaling pathways:

*Canonical signaling pathway* of plasmatic membrane TLRs involves myeloid differentiation primary response 88 (MYD88), which activates nuclear factor-kappa B (NF-κB) by a protein complex with kinases that recruit tumor necrosis factor receptor associated factor 6 (TRAF6) and leads to proinflammatory cytokines production, such as interleukin-6 (IL-6), interleukin-1β (IL-1β) and α, tumor necrosis factor (TNF), interleukin-8 (IL-8) and interleukin-18 (IL-18). The final result of this pathway is a cell survival profile [7]. NF-κB canonical activation after TLR signaling is constituted by the heterodimerization of p65 and p50 subunits. After activation of the IκB kinase, IκB is phosphorylated, ubiquitinated, and degraded by the proteasome that enables NF-κB translocation to nucleus, triggering transcriptional activity [8, 9]. Several of such activated transcripts lead to the upregulation of positive regulators for cell cycle as cyclin D1, with a fundamental role in cellular division and DNA synthesis [10], c-Myc, responsible for enhancing expression of genes related to proliferation [11, 12], c-Jun [13], and serum response factor (SRF) [14, 15].

*Pathway through TLR2 dimerization with TLR1 or TLR6*. TLR2 can form a heterodimer complex with both TLR1 and TLR6 when activated. The heterodimerization of TLR2 with TLR1 or TLR6 expands the ligand spectrum, enabling the innate immune system to recognize different structures of pathogens associated molecular patterns. The downstream signal pathways remain the same for both heterodimers [16].

*Pathway through TLR4 endocytosis*, activating TIR-domain-containing adapter-inducing interferon-β (TRIF), with interferon type 1 response [17].

*Pathway through TLR4 activating ripoptosome complex formation*, composed of receptor-interacting serine/threonine kinase 1 and 3 (RIPK1/3) and mixed lineage domain kinase like pseudokinase (MLKL), tumor necrosis factor receptor type 1-associated death domain protein (TRADD), and fas-associated protein with death domain (FADD). Ripoptosome activation can lead to apoptosis when caspase 8 (CASP8) is present, or to necroptosis when it is absent. TLR5 and TLR2 are indirectly involved in ripoptosome activation by TNF secretion [18–20].

*Pathway through TLR4*, *TLR2*, *and TLR5 forming inflammasome complex*, with the production of Caspase-1 and the proinflammatory cytokines IL-1β and IL-18. The inflammasome complex is constituted by NLRP3, PYCARD, and procaspase 1 [21, 22].

The present study was performed in astrocytoma, a brain tumor with an astrocytic phenotype. The 2007 World Health Organization (WHO) Classification of Tumors of the Central Nervous System (CNS) divided astrocytomas in four malignant grades, I to IV. Glioblastoma (GBM; grade IV astrocytoma) is the most frequent malignant CNS tumor, with a median overall survival of 15 months under current standard of care treatment, which consists of total macroscopic surgical resection combined with radiotherapy and chemotherapy with the alkylating agent, temozolomide [23, 24].

With the onset of next generation sequencing, it was possible to assess the molecular landscape characterizing GBMs. In an attempt to better elucidate the involved molecular tumorigenic processes, and to better guide the adjuvant therapeutic strategies, three main molecular patterns, associated to somewhat distinct clinical outcomes, were identified: 1) the proneural subtype with molecular markers related to progenitor neuronal cells; 2) the classical subtype with markers of proliferative cells; and 3) the mesenchymal subtype with markers of epithelial-mesenchymal transition. Patients exhibiting molecular markers for the mesenchymal subtype presented poor prognosis and the worst response to standard of care, and proneural markers showed better prognosis [25–28]. The result of this initiative prompted a new WHO classification, in 2016, which partially includes molecular aspects of GBMs as segment of its official characterization [29].

This study aims to analyze: **1**) TLR (1, 2, 4, 5, and 6) expressions in WHO grades II to IV (diffusely infiltrative) human astrocytomas and in GBM molecular subtypes, **2**) correlation of TLRs expression levels, and **3**) the presence of these receptors in GBM cell lines.

## Materials and methods

### Tumor samples and ethical statement

The studied cases were composed of 140 astrocytoma grade II-IV samples, collected during therapeutic surgical intervention in the Neurology Department of the Hospital das Clinicas of University of Sao Paulo School of Medicine, by the institutional neurosurgery group. The cases were stratified according to the WHO classification as: 22 non-neoplastic (NN) cases from epilepsy surgery, 26 astrocytoma grade II (AGII) cases, 18 astrocytoma grade III (AGIII) cases, and 96 astrocytoma grade IV (GBM) cases, this cohort has been previously published [30, 31]. The procedures were performed with informed and approved consent according to the Institutional Ethical Committee guidelines at the Hospital das Clinicas of University of Sao Paulo School of Medicine (691/05). The present study was approved by the same institution (059/15) to use the biorepository.

### Sample preparation and RNA extraction

The samples were macrodissected and frozen in liquid nitrogen right after the surgical removal, then cryosectioned for RNA extraction. For the tumor tissue analysis, a 6 µm thick section and hematoxilin-eosin staining was made to guarantee the absence of necrotic, gliosis, non-neoplastic areas, and more than 80% of tumor cells in all tumor tissue specimens [32, 33].

The RNA extraction was accomplished by the RNeasy Mini Kit (Qiagen, Hilden, Germany) following the manufacturer instructions. The RNA concentration and purity were evaluated by NanoDrop, and 1.8–2.0 values for 260 nm and 280 nm absorbance ratios were considered satisfactory. RNA quality was checked by electrophoresis in agarose gel. Reverse transcription was performed with 1 µg of RNA treated with DNase I (FPLC-puro, GE Healthcare, Uppsala, Sweden), and amplified with random primers and oligodT oligonucleotides, RNase inhibitor, and the SuperScript III reverse transcriptase (Thermo Fisher Scientific, Carlsbad, CA), following the manufacturer instructions. Finally, the cDNA was treated with RNase H (GE Healthcare, Uppsala, Sweden), diluted in TE (Tris/EDTA) buffer and stored at -20°C for posterior use by quantitative real-time PCR (qRT-PCR) analysis.

### Quantitative real-time PCR

*TLR1*, *TLR2*, *TLR4*, *TLR5*, and *TLR6* mRNA levels were evaluated by qRT-PCR, using Power SYBR Green. The results were normalized with the geometrical mean of three reference genes for each sample, as previously described [34]: hypoxanthine phosphoribosyltransferase (*HPRT*), glucuronidase beta (*GUSB*), and TATA box-binding protein (*TBP*). The primers were designed to amplify 80-120pb, with melting temperature around 60°C and synthesized by Exxtend (Campinas, Brazil) and IDT (Coralville, IA). The primers were designed as described in Table 1.

**Table 1:** Used primer sequences.

**Gene**	**forward primer (5’-3’)**	**reverse primer (5’-3’)**
*TLR1*	*GGCACCCCTACAAAAGGAATC*	*GATAATGGCAAAATGGAAGATGCT*
*TLR2*	*TGTGGGTTGAAGCACTGGAC*	*TTGCGGTCACAAGACAGAGAAG*
*TLR4*	*TTTATCCAGGTGTGAAATCCAGAC*	*TCCAGAAAAGGCTCCCAGG*
*TLR5*	*CATACTCCTGATGCTACTGACAACG*	*GCAGATGAGAGTAGGGAAGTCCA*
*TLR6*	*AAACGGCACATTCTTCCACAA*	*TTTGTCGTTGTTGTTACTGTGGTTG*
**Reference gene**		
*HPRT*	*TGAGGATTTGGAAAGGGTGT*	*AGCACACAGAGGGCTACAA*
*GUSB*	*GAAAATACGTGGTTGGAGAGCTCATT*	*CGAGTGAAGATCCCCTTTTTA*
*TBP*	*AGGATAAGAGAGCCACGAACCA*	*CTTGCTGCCAGTCTGGACTGT*

Primers were optimized to the minimum concentration for the minor cycle threshold (Ct), the maximum amplification efficiency, and minimum nonspecific amplifications. The mix was composed by cDNA (3 µl), Power SYBR Green PCR Master Mix (Thermo Fisher Scientific, Carlsbad, CA) (6 µl), and the reverse and forward primers (3 µl of each). The qRT-PCR was done in duplicate using the ABI Prism 7500 (Thermo Fisher Scientific, Carlsbad, CA) following the protocol: 2 minutes at 50°C, 10 minutes in 95°C, and 40 cycles of 15 seconds at 95°C, and 1 minute at 60°C. The expression values were assessed by the formula 2^-ΔCt^ as ΔCt is: the Ct of analyzed gene–geometric mean Ct of the reference genes.

### Immunofluorescence

The presence of TLR proteins in tumor cells was analyzed by immunofluorescence. A172 and U87MG human GBM cell lineages were acquired from ATCC and authenticated by short tandem repeats (STR) analysis using GenePrint 10 System (Promega, Madison, WI). Cells were cultured in monolayer with DMEM medium (Dulbecco’s Modified Eagle’s Medium, (Thermo Fisher Scientific, Carlsbad, CA), 10% fetal bovine serum and 100 µg/ml streptomycin and 100 IU/ml penicillin.

Cells were fixed with methanol and acetone (1:1), the membrane was permeabilized with Triton-X-100 (0.1%), and, to avoid unspecific reactions, the cells were treated with 2% bovine serum albumin. The primary antibodies anti-TLR1 (ab180798, rabbit polyclonal, 1:400 diluted), anti-TLR2 FITC-conjugated (AB_945852, ab59711, mouse monoclonal, 1:50 diluted), anti-TLR4 (AB_446735, ab22048, mouse monoclonal 1:200 diluted), anti-TLR5 (AB_793183, sc-57461, mouse monoclonal, 1:50 diluted, Santa Cruz, CA), and anti-TLR6 (AB_2205406, ab37072, rabbit polyclonal, 1:50 diluted) (Abcam, Cambridge, UK) were incubated overnight at 4ºC. The secondary antibodies (Thermo Fisher Scientific, Carlsbad, CA) goat anti-Rabbit IgG H&L (Alexa Fluor 568) and goat anti-Mouse IgG H&L (Alexa Fluor 568 and 488) were incubated for one hour, and nuclei were stained with DAPI (Thermo Fisher Scientific, Carlsbad, CA). The preparations were analyzed in confocal microscopic Zeiss 510 LSM META and Zeiss 780-NLO (Thornwood, NY). The retrieved images were analyzed by Image J/Fiji [35].

### Immunohistochemistry

Paraffin embedded tissue of five representatives cases of GBM, and five non-neoplastic brain tissue from surgical epilepsy cases were stained for the five TLRs, Iba1, CD68 and GFPA by immunohistochemistry using the Novolink kit (Novolink; Novocastra, Newcastle-upon-Tyne, UK), following the manufacture guide. The sections were processed to antigen retrieval, by citrate buffer (10mM, pH6.0) for 3 minutes at 122ºC, using an electric cooker (BioCare Medical, Walnut Creek, USA). After protein blocking, the tissue was incubated with antibodies: anti-TLR1(ab180798, rabbit polyclonal, 1:800 diluted), anti-TLR2 (AB_307008, ab9100, mouse monoclonal, 1:50 diluted), anti-TLR4 (AB_446735, ab22048, mouse monoclonal 1:800 diluted), anti-TLR5 (AB_793183, sc-57461, mouse monoclonal, 1:500 diluted, Santa Cruz, CA), anti-TLR6 (AB_2205406, ab37072, rabbit polyclonal, 1:200 diluted) (Abcam, Cambridge, UK), anti-CD68 (AB_2687454, M0718, mouse monoclonal, 1:1600 diluted, DAKO, CA), anti-IBA1 (AB_2224403, ab15690, mouse monoclonal, 1:700 diluted, Abcam, Cambridge, UK), anti-GFAP (AB_10013382, Z0334, rabbit polyclonal, 1:600, DAKO, CA) at 20° for 16 hours. The reaction performed by the kit uses diaminobenzidine, and for nuclear staining Harris hematoxylin was used. To obtained optimal dilution tonsil sections was used. The immunoreactions for the five TLRs were analyzed according to a semi-quantitative score system considering the intensity of staining (0: negative, 1: weak, 2: moderate and 3: strong), and percentage of immune-positive cells (0: no cells stained, 0,5: 1–10%, 1: 10–25%, 2: 26–50% 3: 51–75% and 4: 76–100%). An immunolabeling score (ILS) was obtained by the product of the intensity of staining and the percentage of stained cells, by two independent investigators (IFM, SKNM), and simultaneous revision were performed to obtain the final score in case the concordance was not achieved. Digital photomicrographs of representative fields were captured and processed using Adobe Illustrator CS6 (Adobe System, San Jose, CA).

### Statistical analysis

The statistical analysis was performed in the program SPSS version 23.0 (IBM, Armonk, NY). The data distribution was evaluated by Kolmogorov-Smirnov test. The non-parametric results were evaluated by Kruskal-Wallis test, followed by post hoc Dunn’s test. The correlations between each two receptors gene expression levels were assessed by Spearman-rho. The associations are considered strong when *r* ≥ 0.7, moderate when 0.4 < *r* > 0.7, and low when *r* ≤ 0.4. Differences were considered significant for *p* < 0.05.

The GBM RNAseq dataset from *The Cancer Genome Atlas* (TCGA - http://cancergenome.nih.gov/) was downloaded from *.rsem.genes.normalized_results address. The data was analyzed by DE-seq and the RPKM values were normalized by the z-score.

## Results

### *TLR1*, *TLR2*, *TLR4*, *TLR5*, and *TLR6* mRNAs were up-regulated in diffuse astrocytomas

**Fig 1:**
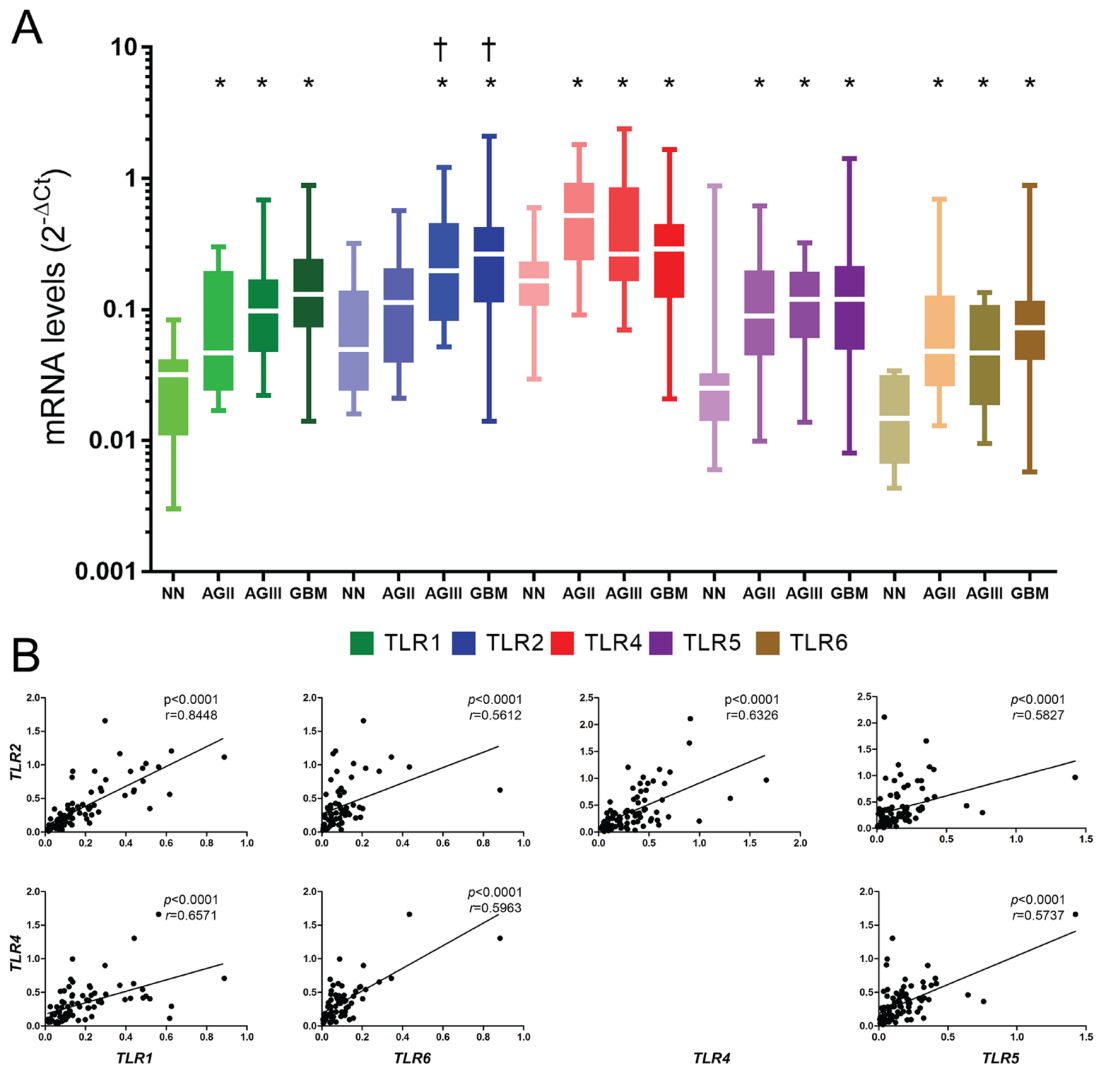
*TLR1*, *TLR2*, *TLR4*, *TLR*5, and *TLR*6 expression levels in astrocytomas of different malignant grades. (A) The analyzed samples consisted of 22 non-neoplastic (NN) cases, 26 astrocytoma grade II (AGII) cases, 18 astrocytoma grade III (AGIII) cases, and 96 glioblastoma (GBM) cases. Data are represented by box and whisker plots, with the median represented by the line in the middle of the boxes, and top and bottom boxes represent the first and third quartiles. qRT-PCR values are normalized by three housekeeping genes (*HPRT*, *GUSB*, *TBP*). For statistical analysis, Kruskal-Wallis and Dunn’s tests were applied, wherein (*) *p* < 0.05 when compared to NN cases and (†) *p* < 0.05 when compared to AGII (Dunn test), all the genes present *p*<0.01 (Kruskal-Wallis). (B) Correlation between *TLR2*-*TLR1*, *TLR2*-*TLR6*, *TLR2*-*TLR4*, *TLR2*-*TLR5*, *TLR4*-*TLR1*, *TLR4*-*TLR5*, and *TLR4*-*TLR6* are demonstrated in GBM cases. Statistical analysis was made by the Spearman-rho correlation, and *p*<0.05 were considered significative.

Genes coding the five receptors, *TLR1*, *2*, *4*, *5*, and *6*, presented higher expression levels in astrocytoma samples compared to non-neoplastic brain tissues, with statistical significance (*p* < 0.05) (Fig 1A). Only *TLR2* expression in AGII did not present any statistical difference compared to NN tissue, in contrast to the statistical difference observed in AGIII and GBM (*p* < 0.05). *TLR1*, *TLR2*, and *TLR6* mRNA expression median values showed an increase in parallel to the increase of malignancy. On the other hand, the median *TLR4* expression in GBM cases was lower than in the other astrocytoma grades (AGII-AGIII).

### Associated *TLRs* expressions were observed in GBM

All TLRs expressions were correlated between themselves in GBM, with statistical significance of *p* < 0.05 (Fig 1B). Expression levels of *TLR2* and *TLR1* presented the strongest correlation (*r* = 0.8448, *p* < 0.0001 by Spearman-rho test).

### Higher *TLR4* and *TLR6* median expression levels were observed in mesenchymal GBM compared to other molecular subtypes

**Fig 2:**
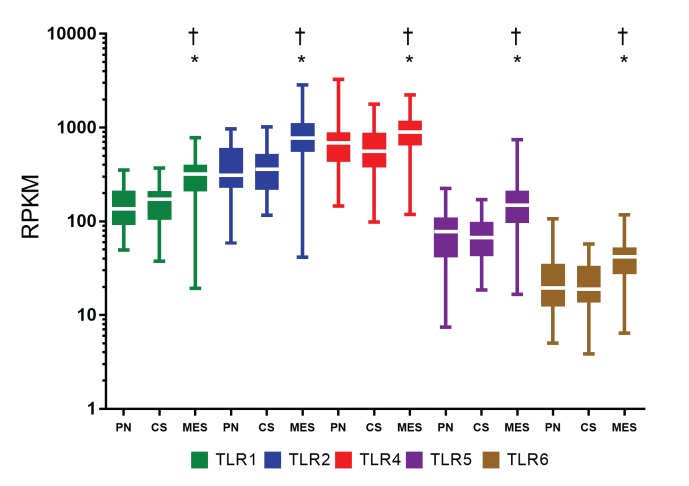
*TLR1*, *TLR2*, *TLR4*, *TLR*5, and *TLR*6 expression levels in GBM molecular subtypes from TCGA dataset. Data are represented by box and whisker plots, with the median represented by the line in the middle of the boxes, and top and botton boxes represent the first and third quartiles. The dataset was divided into 37 proneural (PN) cases, 40 classical (CS) subtype cases, and 55 mesenchymal (MES) subtype cases, in which (*) and (†) are *p* < 0.05 when mesenchymal group was compared to proneural cases and to classical cases, respectively (Dunn’s test) and *p*<0.01 (Kruskal-Wallis).

Mesenchymal GBM subtype presented higher *TLR4* and *TLR6* mRNA median levels than other molecular subtypes in our cohort (S1 Fig.) [36]. However, given the reduced sample number, particularly for the proneural and mesenchymal GBM cases in this cohort, statistical significance was not achieved. Therefore, we performed a similar analysis in molecular GBM subtypes of TCGA public dataset. In this larger cohort, all five TLRs presented higher expression in mesenchymal subtype with statistical significance compared to proneural and classical subtypes (Fig 2).

### *TLR1*, *TLR2*, *TLR4*, *TLR5*, and *TLR6* were present in tumor cell lines

**Fig 3:**
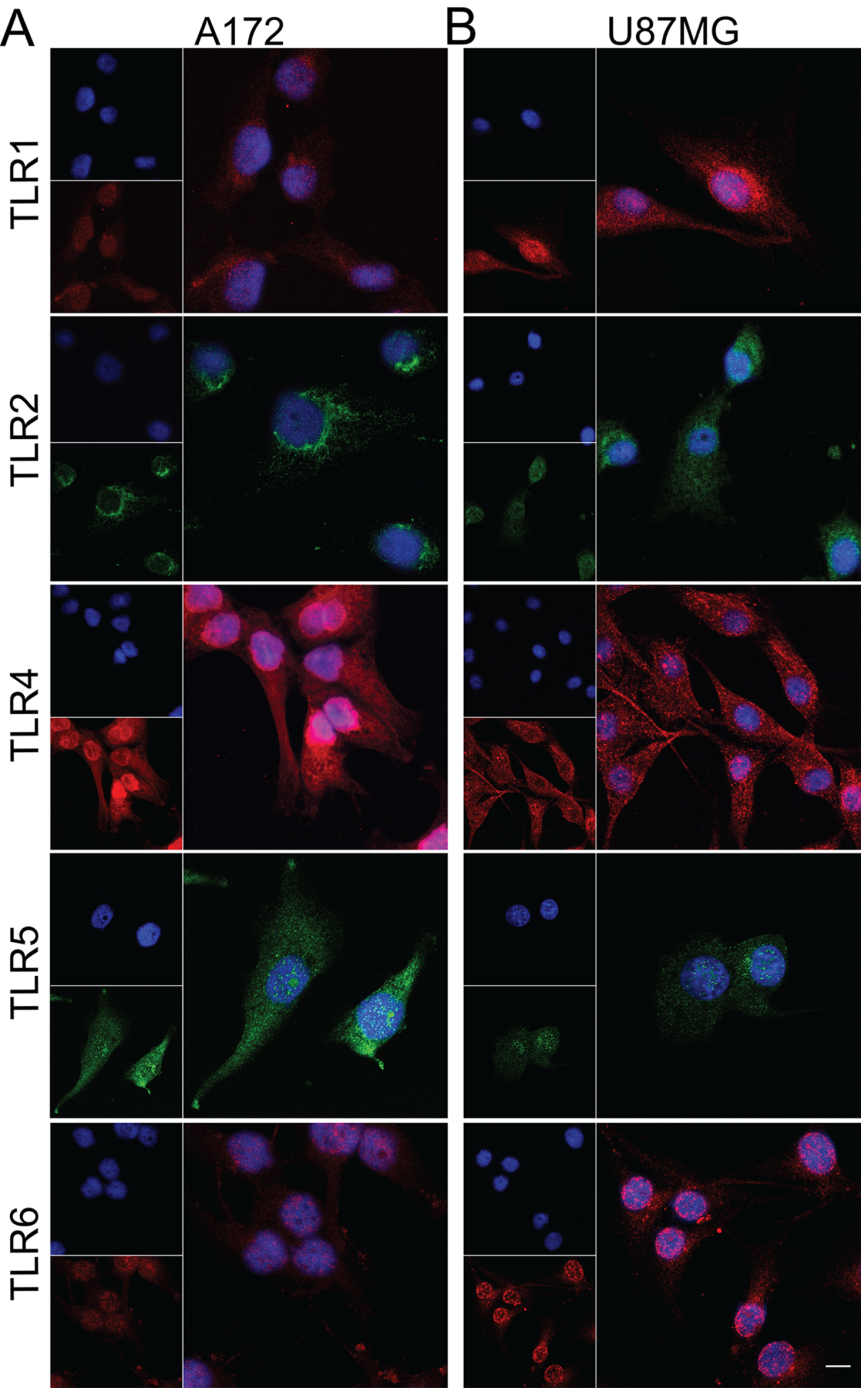
Immunofluorescence of TLR1, TLR2, TLR4, TLR5, and TLR6 in GBM cell lines. A172 (A) and U87MG (B). TLR1, TLR4, and TLR6 are stained in red, TLR2 and TLR5 in green, and nuclei in blue by DAPI. The presence of all five TLRs was detected in both cell lines. Expression of TLR5 was more intense in A172 compared to U87MG. TLR4 and TLR5 positivity were detected in both tumor lineage cells nuclei. Magnification of 400x.

The five TLR proteins were identified in tumor cells, in both GBM cell lineages with mutational status consistent for the mesenchymal subtype (U87MG and A172): RB transcriptional corepressor (*RB1*) missense mutation in A172, and neurofibromin (*NF1*) missense and deletion mutation in U87MG [37]. For the immunofluorescence observation (Fig 3), TLR5 presented lower protein expression in U87MG compared to A172. The evaluation of these *TLR* mRNA levels by qRT-PCR corroborated their presence in both cell lineages; *TLR5* mRNA expression level was not detected in U87MG cells by qRT-PCR (S2 Fig.).

### *TLR1*, *TLR2*, *TLR4*, *TLR5*, and *TLR6* protein presence in GBM tissue

**Fig 4:**
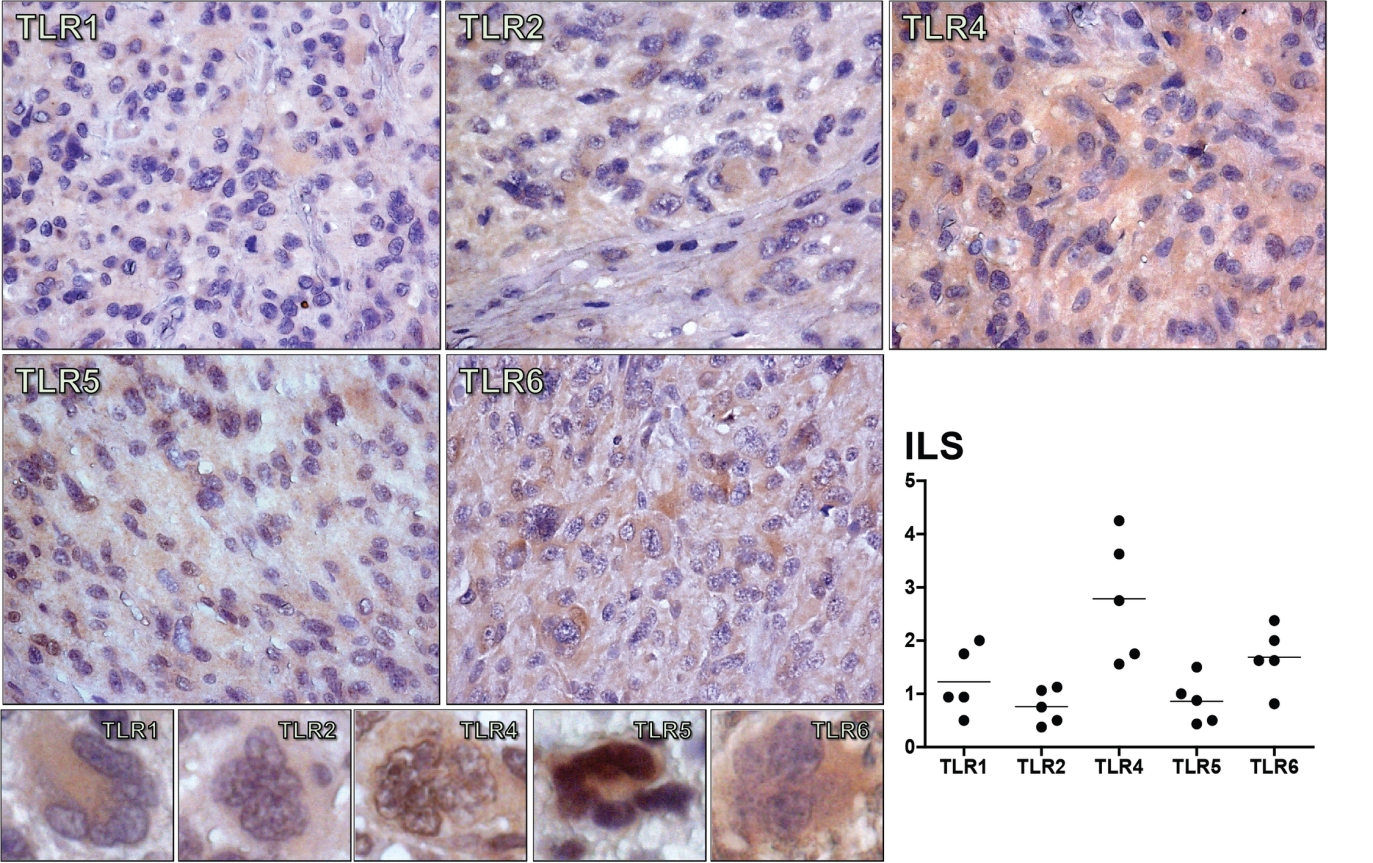
Imunohistochemistry for TLR1, TLR2, TLR4, TLR5, and TLR6 in GBM cases. Positive immunolabelling of GBM tumor cells for TLRs are demonstrated at 600x magnification of tumor tissues, and at 800x magnification in a multinucleated GBM tumor cell. The distribution of the Immunolabeling score (ILS) for these TLRs in five GBM cases was presented as a dispersion graph, where the black dots represent the mean ILS obtained by the two independent investigators for each individual tumor case, and the horizontal bar represent the mean ILS for each receptor. TLR4 and TLR5 positive staining were detected in tumor nuclei.

The five TLRs proteins were detected in phenotypically GBM cells in all cases, including on the multinucleated GBM cells, corroborating the findings observed in the GBM cell lineages. The TLR4 protein expression was the highest among the five studied TLRs. Interestingly, TLR4 and TLR5 protein expressions were also observed in tumor cell nuclei, as observed in the immunofluorescence preparations of the GBM cell lineages (Fig 4). The presence of few microglia in the GBM tumor sample were detected by IBA1 staining and few macrophages with CD68, which did not overlap with the TLRs positive tumor cells. The positive reaction for GFAP was consistent with the glial origin of the analyzed GBM tumor samples (S3 Fig). Non-neoplastic brain samples were also stained for TLRs. As expected, the positivity for these receptors were observed in neurons and the lowest expression was for TLR2. Similar to the staining pattern in tumor cells, TLR5 positivity was detected in neuron nuclei (S3 Fig).

### *In silico* exploration of TLR signaling pathway

**Fig 5:**
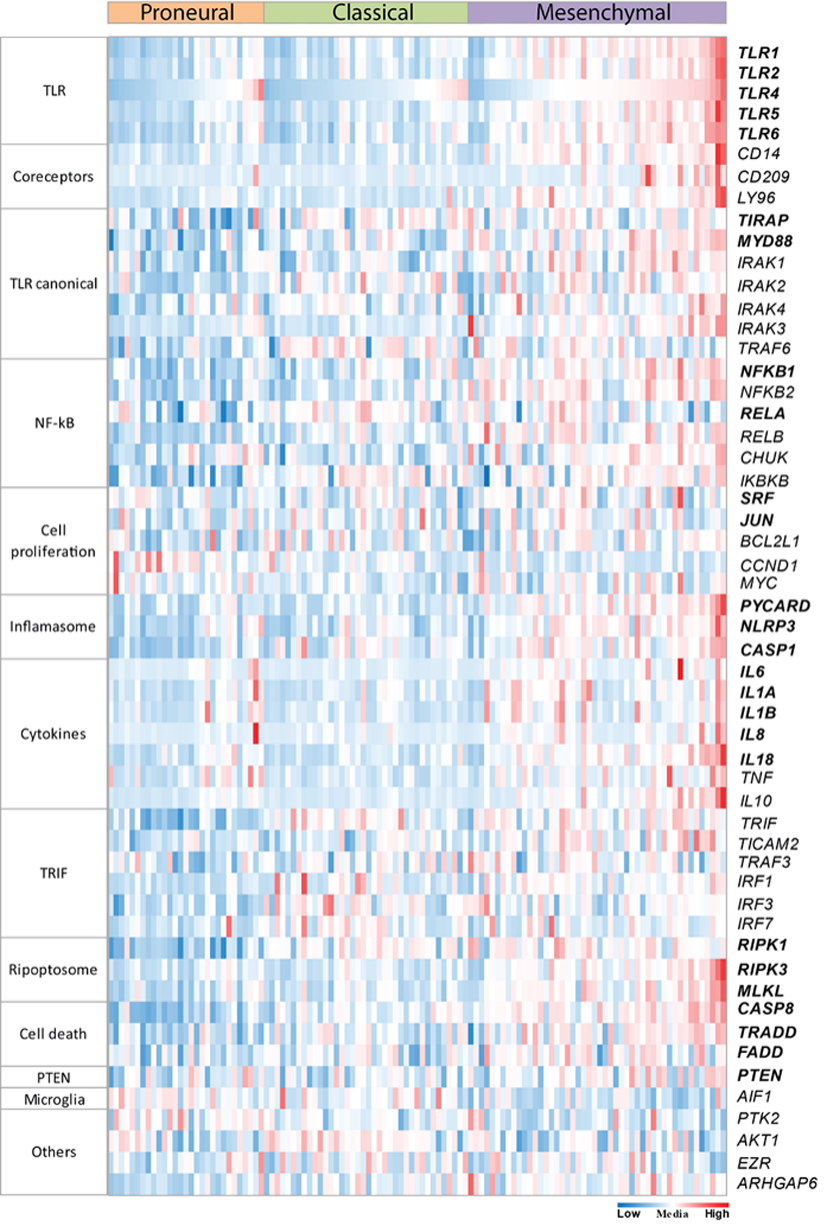
Heatmap with major genes of the TLR signaling pathways from the TCGA dataset. RPKM gene expression levels are normalized by z-scores, and comparatively up-regulated RNA expression values are presented in red and down-regulated values in blue. Mean values are in white. TLRs downstream signaling pathways: canonical, ripoptosome, and inflammasome pathways are activated in mesenchymal GBM subtype. Genes of unrelated pathways were added to show their randomic expression levels, including a microglia marker.

A heatmap including selected target genes of TLR signaling pathways was built from the TCGA RNAseq dataset. The RPKM values (S4 Fig.) were normalized by the z-score values for each gene to allow comparative analysis among them (Fig 5). *In silico* analysis highlighted the higher expression of TLRs in mesenchymal subtype relative to proneural and classical subtypes (S4 Fig.), and pointed out a downstream upregulation through MYD88/TIRAP and NF-κB, ending with increased expression levels of genes that code for cytokines and genes related to cell proliferation. Particularly, expression levels of genes that code for IL-8, IL-6, IL-1α, IL-1β, and IL-18 were differentially increased in the mesenchymal GBM subtype compared to the other two subtypes (S4 Fig.). Moreover, *RELA*, which codes for the nuclear subunit of NF-κB, presented positive correlations (*p* < 0.05) by Spearman-rho test with expression levels of cell proliferation genes: *JUN* (*r* = 0.422 and *r* = 0.536) and *SRF* (*r* = 0.462 and *r* = 0.577) and in mesenchymal and classical subtypes, respectively. In contrast, the gene expressions related to interferon type I response presented low or undetermined z-score values (not shown). Interestingly, expression levels of genes involved in ripoptosome signaling, such as *RIPK3* and *MLKL*, were also comparatively increased in mesenchymal GBM subtype, and their values correlated to *TLR2* (*r* = 0.795 and *r* = 0.661, respectively), and to *TLR4* (*r* = 0.575 and *r* = 0.360, respectively). Of note, *PYCARD*, *NLRP3* and *CASP1*, which participate in the inflammasome pathway, presented higher expression levels in mesenchymal subtype of GBM compared to the other two subtypes (S4 Fig.).

## Discussion

In the present study, higher expression levels of *TLR1*, *TLR2*, *TLR4*, *TLR5*, and *TLR6* were demonstrated in human diffusely infiltrating astrocytomas (grades II to IV) in comparison to non-neoplastic brain tissue. Moreover, an associated expression of these receptors were observed in GBM cases, suggesting their role in tumor aggressiveness. The presence of TLR4 has been previously described in GBM, particularly in CD133^+^ tumor stem cells [38, 39], and also in GBM cell lineages A172, U87MG [3, 40, 41], and U251 [42]. Such TLR4 positivity conferred a proliferative phenotype to these tumor cells [38, 42]. Furthermore, the presence of TLR2 was also detected in murine GL261 glioma cell line, and the activation of this receptor leads to an invasive and migratory profile of the tumor cells [43]. We further demonstrated herein the presence of TLR1, TLR2, TLR5, and TLR6 in U87MG and A172 cell lineages and in human GBM specimens, at gene and protein expression levels. TLR4 was the most expressed receptor in both cell lineages and tumor specimens. Interestingly, TLR4 and TLR5 positivity were detected in the tumor cell nuclei by immunofluorescence in cell lineages and by immunohistochemistry in tumor specimens. However, such observation need confirmation in other cohorts before speculating their possible role in this localization (Figs 3, 4 and S2). In spite of both cell lineages presenting somatic mutation profiles of mesenchymal molecular subtype of GBM, they present distinct expression profiles of these TLRs. U87MG cells showed higher expression of *TLR1*, *TLR2*, and *TLR4* compared to A172, in contrast to lower expression of TLR5 (Figs 3 and S2). Such heterogeneity of these TLRs expression distributions was also observed in human astrocytoma samples from our cohort and in the TCGA dataset, particularly among GBMs, where higher expression levels were detected in mesenchymal subtype (Figs 1, 2, 5 and S1). To analyze the impact of these differential TLRs expressions among the molecular subtypes of GBM, we built a heatmap with the expression levels of the genes involved in pathways related to TLRs from the TCGA dataset (Fig 5). Interestingly, this approach showed clearly the downstream activation of the canonical, ripoptosome, and inflammasome pathways, related to the upregulation of the TLRs, particularly in the mesenchymal subtype of GBM. The end targets of these pathways, including cytokines (*IL1A/B*, *IL6*, *IL8*, *IL18*) and genes related to cell proliferation (*JUN*, *SRF*), were upregulated, suggesting that the activation of these TLRs leads to tumor growth. In fact, activation of the TLR canonical pathway through TIRAP-MYD88 was related to NF-κB (*RELA*) upregulated expression levels and positive correlation with *JUN*, and *SRF*, transcription factors implicated in cellular proliferation. The role of NF-κB in tumor growth has been demonstrated in a mouse model of GBM [44] and in pulmonary tumor cells [45]. Additionally, it has been reported that such a proliferative response may be time- and dose-dependent [46]. Therefore, tumor inflammatory microenvironment may contribute to distinct NF-κB pulsation-determining tumor cell behavior in each specific condition. The end targets of this pathway, IL-6, IL-1α and IL-1β, and IL-8 cytokines, presented increased expression in the mesenchymal subtype, and they have also been associated to tumor malignancy and tumor cell migration [22, 47–49]. *IL1B* and *IL18* are also end targets of the inflammasome pathway, which were upregulated in the mesenchymal subtype. On the other hand, the increased expression of *RIPK3* and *MLKL* may suggest the upregulation of the ripoptosome pathway, and may allow the link with the presence of necrosis, one of the characteristics of GBM. In a rat glioma model, apoptosis was induced by activation of CASP8 and inhibition of RIPK1/RIPK3 complex [19, 50, 51].

The expression of *CASP8* was increased in the mesenchymal subtype of GBM; however, increased apoptosis is not expected and, therefore, avoidance of apoptosis should be under additional modulation. Moreover, according to the present *in silico* analysis, the interferon type I response by TLR4 endocytosis was unlikely.

**Fig 6:**
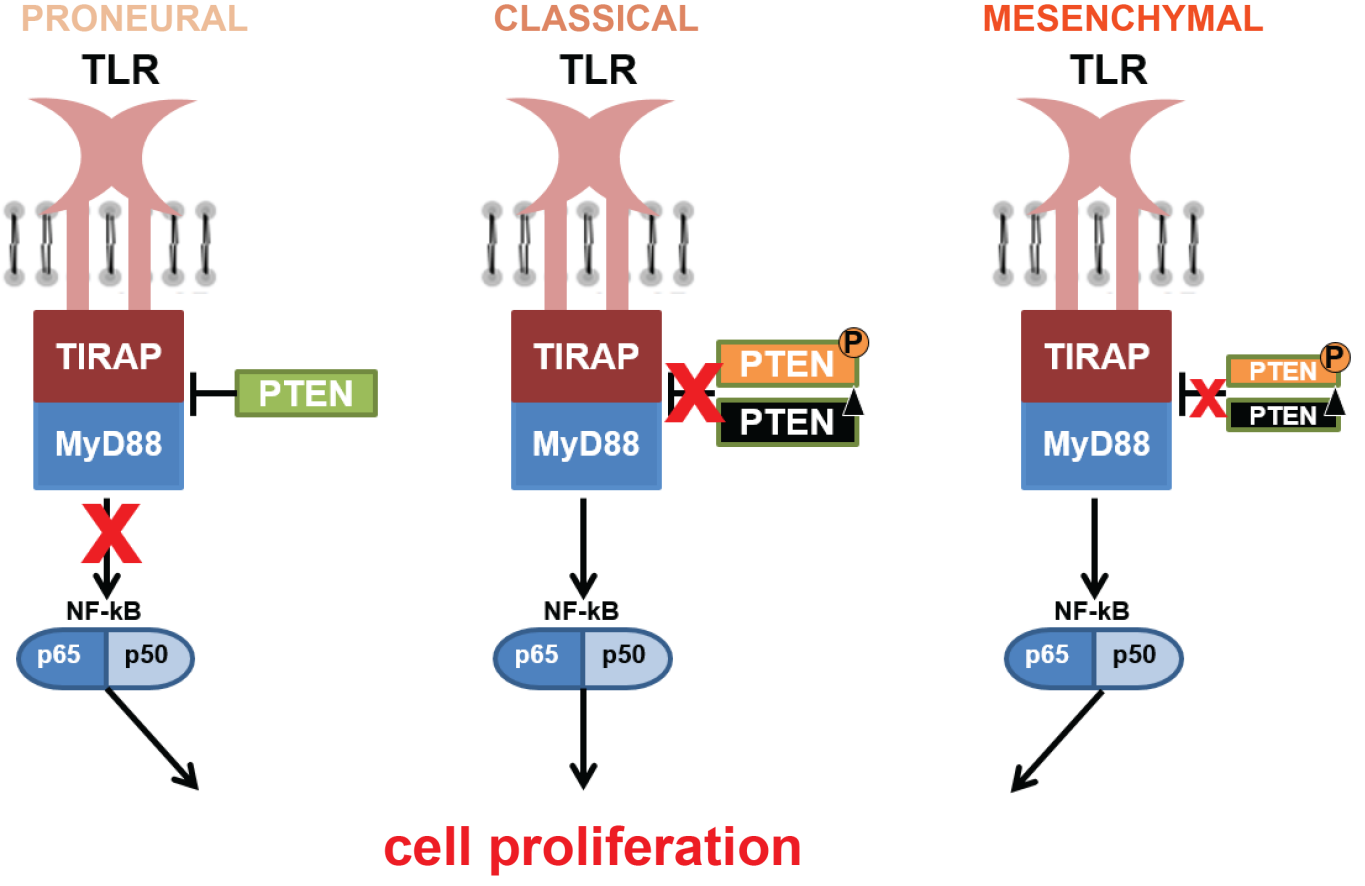
Schematic proposition of the TLR canonical signaling pathway through TIRAP by PTEN regulation. PTEN alterations may lead to upregulation of TLR signaling pathway and may increase tumor cell proliferation. Loss of PTEN repressor role by its deletion or phosphorylation, as may occur more frequently in classical subtype, may activate downstream TLR canonical pathway through the decreased inhibition over TIRAP. This pathway through TIRAP-PTEN may not be the major mechanism in mesenchymal subtype, and the integrity of PTEN may inhibit TIRAP and then not activate this pathway in proneural subtype.

Another hypothesis for the proliferative profile and TLR signaling in GBM is the pathway involving PTEN, which has been shown to regulate TIRAP and TLR4 internalization [52, 53], in addition to its classic suppressor role in the PI3K-AKT-mTOR pathway [54]. Loss of function of PTEN may occur by its deletion or phosphorylation [27, 55]. Our previous analysis of PTEN status in our GBM cohort demonstrated 24.32% and 26.66% of *PTEN* deletion, and 59.45% and 66.66% of PTEN phosphorylation in classical and mesenchymal subtypes, respectively (S5 Fig.) [36, 55], demonstrating more prevalent *PTEN* loss of function in the classical subtype. These PTEN alterations may lead to a decreased inhibition over TIRAP (Fig 6) and consequent TLR canonical pathway activation. In B cells, the absence of PTEN showed an increased in NF-κB activity after TLR4 stimulation [56]. Additionally NF-κB negatively regulate PTEN [57, 58]. Accordingly, in the classical subtype, loss of PTEN may also be implicated in tumor cell proliferation through TLR canonical activation (Fig 5). Actually, our analysis of the TCGA dataset showed significant correlation between *TIRAP* and *PTEN* expression levels among the classical GBM subtype (*r* = 0.330, *p* < 0.05, Spearman-rho test). Interestingly, a negative correlation was found between them in the proneural subtype (*r* = -0.382, *p* < 0.05), which presents better prognosis among the three analyzed GBM subtypes.

In summary, increased cell membrane *TLR1*, *TLR2*, *TLR4*, *TLR5*, and *TLR6* expressions were demonstrated in astrocytomas compared to non-neoplastic brain tissue, mostly in GBM group, and particularly in the mesenchymal subtype. *In silico* exploration of the putative activation pathways by these upregulated TLRs, the canonical and inflammasome pathways in the mesenchymal subtype and the PTEN-TIRAP signaling in the classical subtype demonstrated their increase and their possible contribution to tumor growth. According to the TCGA dataset the microglia compartment based on *AIF1* expression level did not parallel the *TLR*s upregulated expressions in mesenchymal subtype, suggesting that the tumor cells themselves may contribute to the increased expression of these receptors.

Further understanding of the particular TLR responses from each tumor compartment, which includes inflammatory cells in addition to tumor cells, will be essential to designing new therapeutic strategies involving TLRs.

## Supporting information

S1 Fig TLRs expression levels in the GBM subtypes of our cohort composed of 14 proneural (PN), 38 classical (CS) and 16 mesenchymal (MES) subtype cases. Horizontal bars indicate the mean value of each group.
(TIF)

S2 Fig *TLRs* expression levels in U87MG and A172 cell lines. A heterogeneity in the distribution of the five *TLRs* is observed between the cell lines, although both GBM cell lines present somatic mutation profiles of mesenchymal subtype. *TLR4* expression is high in both cell lines, whereas *TLR5* is undetectable in U87MG cell line.
(TIF)

S3 Fig TLRs protein expression in non-neoplastic cases and microglia and macrophage detection in GBM sample. Immunohistochemistry for a representative non-neoplastic case stained for TLR1, TLR2, TLR4, TLR5, TLR6 and GFAP for glial cell identification. Presence of few microglia in the same GBM sample of Fig 4 was observed by IBA1 staining, and also few macrophages was detected by CD68 staining. GFAP positivity was shown in the GBM tumor sample confirming the glial origin of the tumor.
(TIF)

S4 Fig Expression levels of the genes participating in the TLR signaling pathways divided by GBM molecular subtypes: Proneural, classical, and mesenchymal from the TCGA RNASeq dataset. The dataset is composed of 37 proneural (PN), 40 classical (CS), and 55 mesenchymal (MES) subtype cases, wherein (*) *p* < 0.05 when compared to proneural cases and (†) *p* < 0.05 when compared to classical cases by Kruskal-Wallis and Dunn’s test. The values were normalized in DEseq.
(TIF)

S5 Fig PTEN. The GBM cohort from our lab was previously analyzed for PTEN mutational and phosphorylation status. The number of cases for each status of PTEN and GBM subtype are presented. In green is the amount of cases presenting wild-type PTEN, in black the deleted *PTEN*, and in orange the Y240-phosphorylated PTEN.
(TIF)
