# Supplementary figures and images for: Plasmatic membrane toll-like receptor expressions in human astrocytomas
Source: PLoS One. 2018 Jun 18;13(6):e0199211. doi: 10.1371/journal.pone.0199211 (PMC6005538; doi:10.1371/journal.pone.0199211)

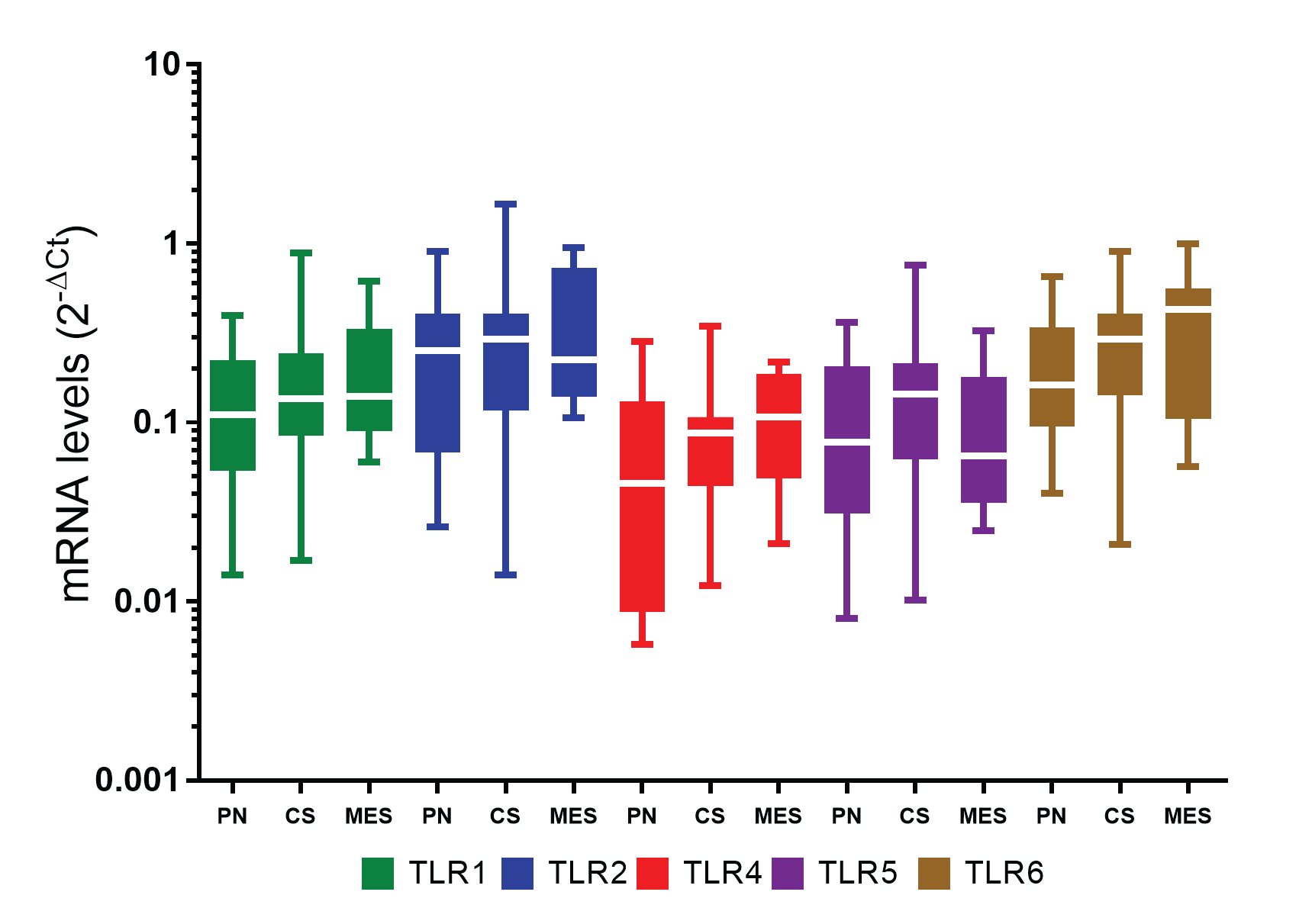

Supplement: S1 Fig — Horizontal bars indicate the mean value of each group. (TIF) [file pone.0199211.s001.tif]

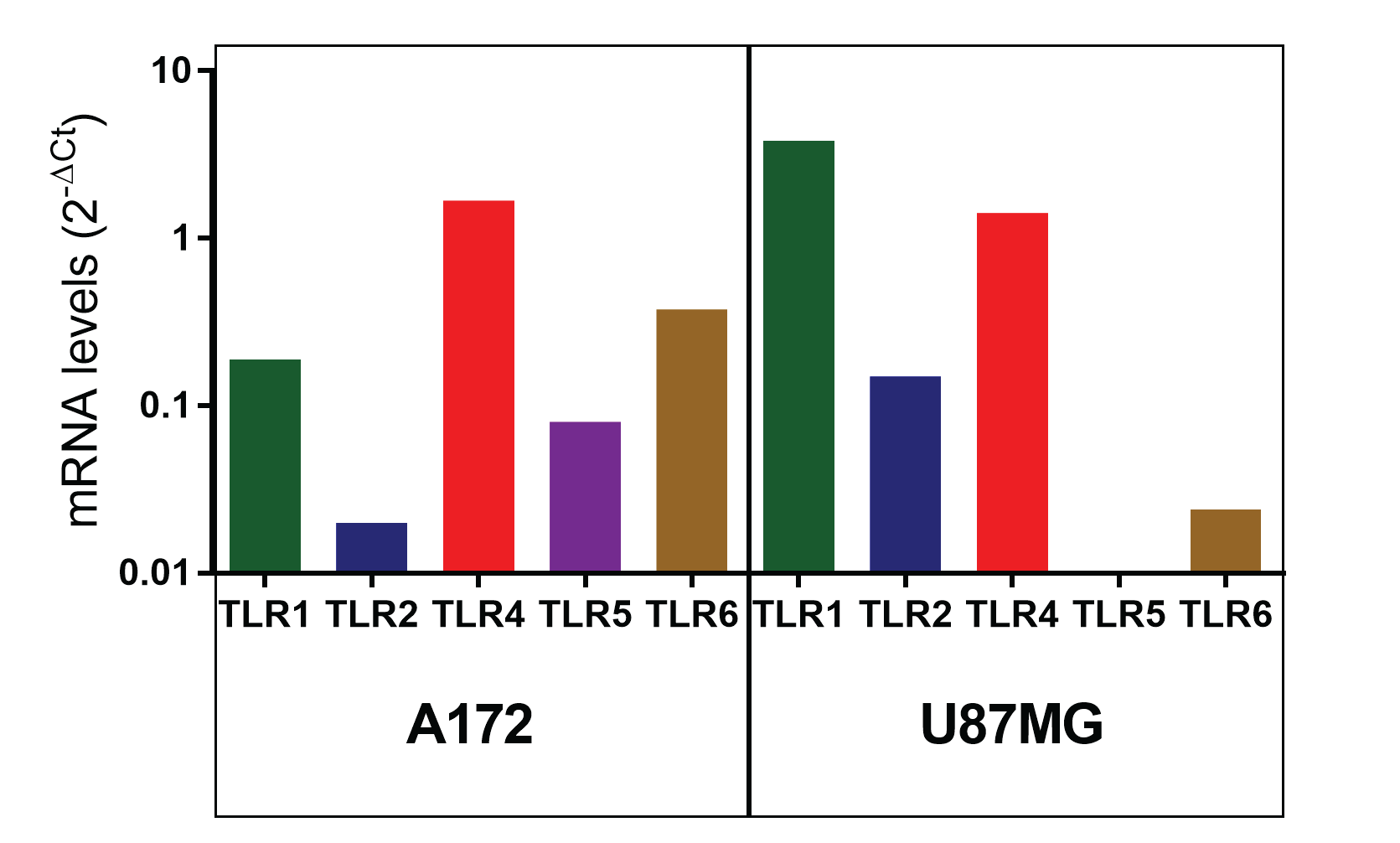

Supplement: S2 Fig — A heterogeneity in the distribution of the five TLRs is observed between the cell lines, although both GBM cell lines present somatic mutation profiles of mesenchymal subtype. TLR4 expression is high in both cell lines, whereas TLR5 is undetectable in U87MG cell line. (TIF) [file pone.0199211.s002.tif]

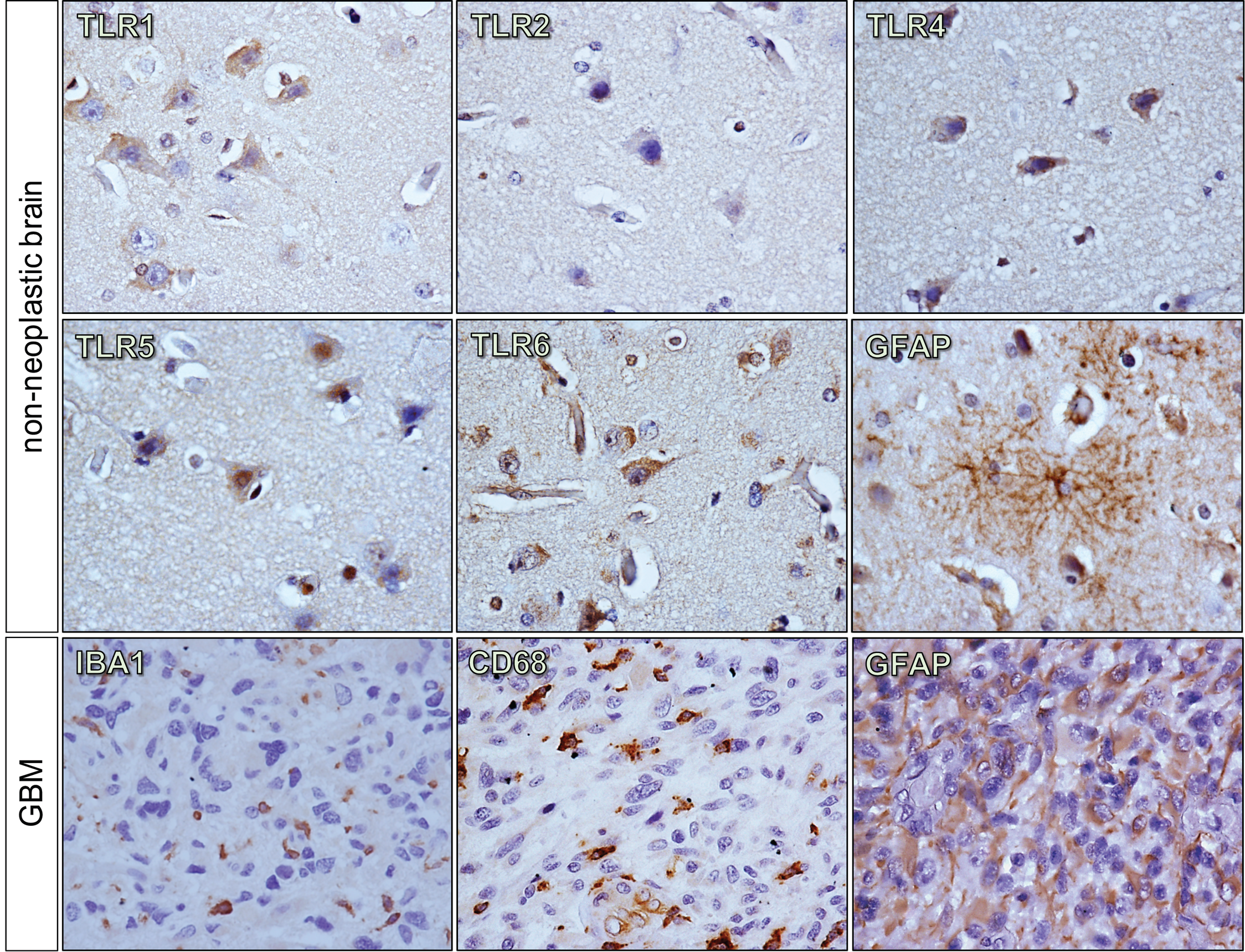

Supplement: S3 Fig — Immunohistochemistry for a representative non-neoplastic case stained for TLR1, TLR2, TLR4, TLR5, TLR6 and GFAP for glial cell identification. Presence of few microglia in the same GBM sample of Fig 4 was observed by IBA1 staining, and also few macrophages was detected by CD68 staining. GFAP positivity was shown in the GBM tumor sample confirming the glial origin of the tumor. (TIF) [file pone.0199211.s003.tif]

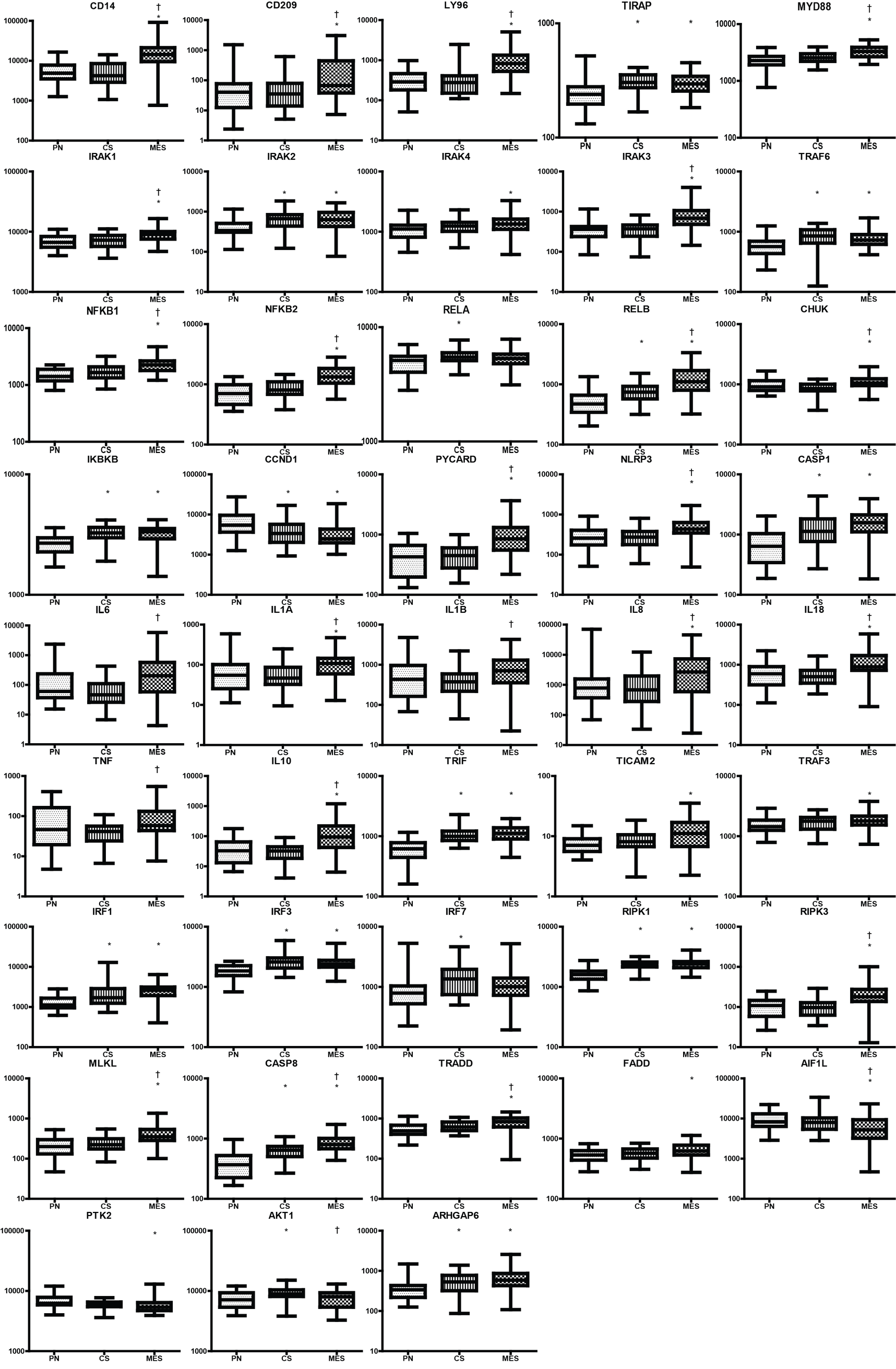

Supplement: S4 Fig — The dataset is composed of 37 proneural (PN), 40 classical (CS), and 55 mesenchymal (MES) subtype cases, wherein (*) p < 0.05 when compared to proneural cases and (†) p < 0.05 when compared to classical cases by Kruskal-Wallis and Dunn’s test. The values were normalized in DEseq. (TIF) [file pone.0199211.s004.tif]

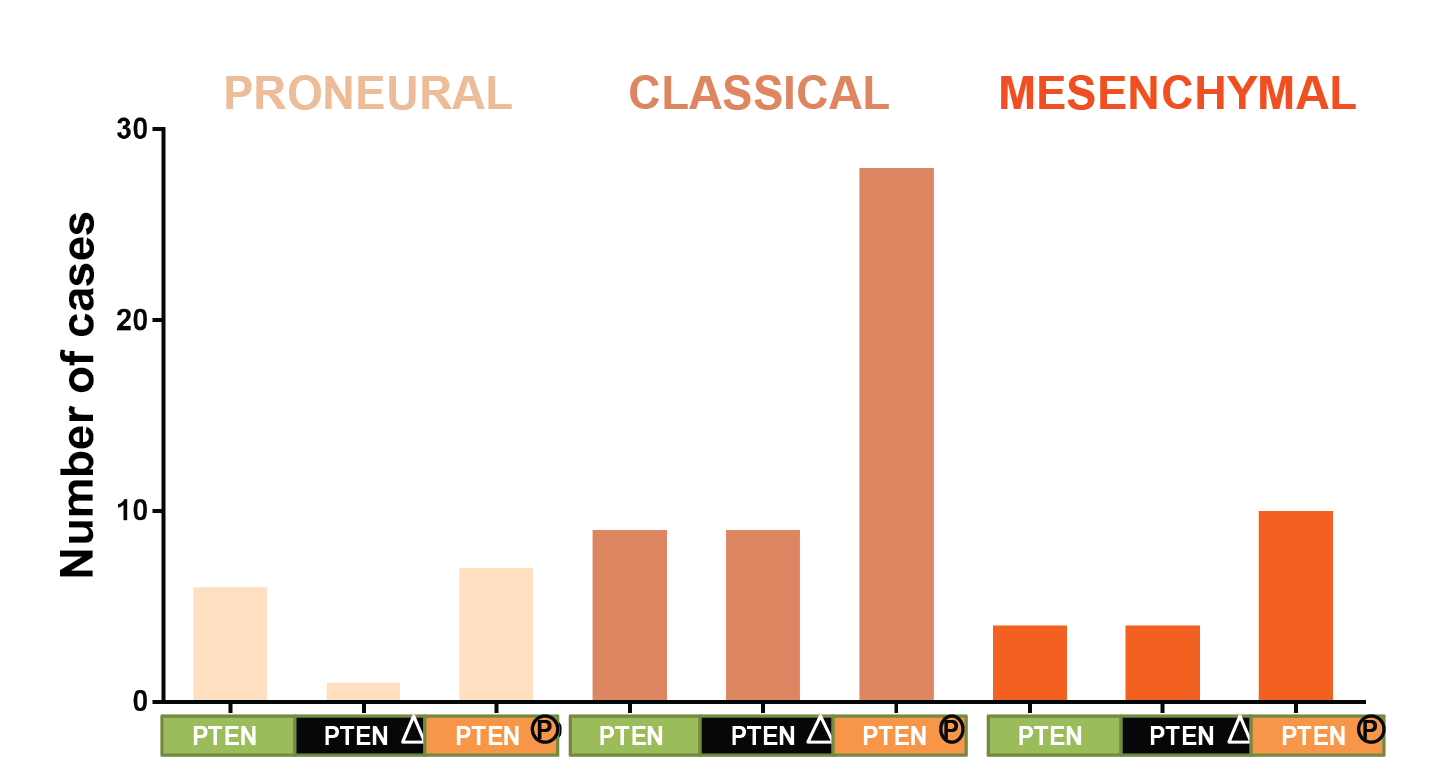

Supplement: S5 Fig — The GBM cohort from our lab was previously analyzed for PTEN mutational and phosphorylation status. The number of cases for each status of PTEN and GBM subtype are presented. In green is the amount of cases presenting wild-type PTEN, in black the deleted PTEN, and in orange the Y240-phosphorylated PTEN. (TIF) [file pone.0199211.s005.tif]
